# Increased risk for hepatitis C associated with solvent use among Canadian Aboriginal injection drug users

**DOI:** 10.1186/1477-7517-7-16

**Published:** 2010-07-19

**Authors:** Souradet Y Shaw, Kathleen N Deering, Ann M Jolly, John L Wylie

**Affiliations:** 1 Centre for Global Public Health, University of Manitoba, R070 Med Rehab Bldg 771 McDermot Avenue, Winnipeg, Manitoba R3E 0T6, Canada; 2 Department of Community Health Sciences, University of Manitoba S113 - 750 Bannatyne Avenue, Winnipeg, Manitoba R3E 0W3, Canada; 3 School of Population and Public Health, University of British Columbia 2206 East Mall, Vancouver, British Columbia V6T 1Z3, Canada; 4 Centre for Communicable Diseases and Infection Control, Public Health Agency of Canada 100 Eglantine Driveway-Tunney's Pasture, Ottawa, Ontario K1A 0K9, Canada; 5 Department of Epidemiology and Community Medicine, University of Ottawa Room 3104-451 Smyth Road, Ottawa, Ontario K1H 8M5, Canada; 6 Department of Medical Microbiology, University of Manitoba 745 Bannatyne Avenue, Winnipeg, Manitoba R3E 0J9, Canada; 7 Cadham Provincial Laboratory, Manitoba Health 750 William Avenue, Winnipeg, Manitoba R3C 3Y1, Canada

## Abstract

**Background:**

Solvent abuse is a particularly serious issue affecting Aboriginal people. Here we examine the association between solvent use and socio-demographic variables, drug-related risk factors, and pathogen prevalence in Aboriginal injection drug users (IDU) in Manitoba, Canada.

**Methods:**

Data originated from a cross-sectional survey of IDU from December 2003 to September 2004. Associations between solvent use and variables of interest were assessed by multiple logistic regression.

**Results:**

A total of 266 Aboriginal IDU were included in the analysis of which 44 self-reported recent solvent use. Hepatitis C infection was 81% in solvent-users, compared to 55% in those reporting no solvent use. In multivariable models, solvent-users were younger and more likely to be infected with hepatitis C (AOR: 3.5; 95%CI: 1.3,14.7), to have shared needles in the last six months (AOR: 2.6; 95%CI:1.0,6.8), and to have injected talwin & Ritalin (AOR: 10.0; 95%CI: 3.8,26.3).

**Interpretation:**

High hepatitis C prevalence, even after controlling for risky injection practices, suggests that solvent users may form closed networks of higher risk even amongst an already high-risk IDU population. Understanding the social-epidemiological context of initiation and maintenance of solvent use is necessary to address the inherent inequalities encountered by this subpopulation of substance users, and may inform prevention strategies for other marginalized populations.

## Background

In developed countries, sexually transmitted infections (STI) and bloodborne pathogens (BBP) disproportionately affect marginalized populations. In the United States, Australia, and Canada the combined impact of poverty, lack of access, and historical and systemic oppression have resulted in overrepresentation of indigenous populations in national HIV/AIDS and STI statistics, especially amongst females and youth[1-6]. Within Canada, injection drug users (IDU) account for a significant proportion of prevalent HIV and other BBP (such as hepatitis C [HCV]) infections, and are an especially important risk group sustaining endemicity of these pathogens within Aboriginal populations[4,7-9]. However, despite progress in, and substantial efforts towards both understanding, and addressing BBP epidemics in Canadian Aboriginal populations[7], the transmission of some BBP, such as HIV and HCV, appear to be growing unabated[10-12]. This paradox has motivated researchers to examine heterogeneity in marginalized subpopulations, with the intention of finding and describing subpopulations that may be at particularly high risk of BBP transmission, as well as the environmental contexts within which they are embedded[13-16].

To this end, solvent abuse has been shown to be a particularly serious and destructive issue affecting Aboriginal populations in Canada, and elsewhere[17-25]. In North America, the lifetime use of solvents has been reported to be as high as 44% in some high-risk groups[26], with some studies finding the prevalence of lifetime use at 17% by the eighth grade[27]. Solvent use is a term broadly applied to the self-administered inhalation of a variety of volatile, psychoactive substances that are found in many common products, including gasoline and adhesive glue[24,28]. Solvent users have elevated rates of negative health outcomes including mental illness[29,30], damage to the central nervous system, heart and lungs [28,31,32], as well as mortality[32,33]. Contributing to its perniciousness, solvents are primarily legal and easily obtainable[18,34]. As well, multiple factors have been identified as being associated with solvent use, including age, sex, ethnicity, education level, co-existing alcohol or other substance use disorders, and child and physical abuse[35-39]. In youth, solvent use has been linked to broader societal issues such as higher school drop-out rates[40], delinquency (including criminal activity)[36,39] and family conflict[39,41]. Salient to this study, an association between chronic solvent use in adolescence and injection drug use among the most marginalized of populations has been demonstrated[20,42-44].

On the treatment side, a particular defining feature of chronic solvent use is that it is typically associated with the most marginalized populations, with, for example higher levels of anti-social behaviour, trauma-exposure and psychiatric morbidities[19,24,45]. In response to the burgeoning need for Aboriginal-specific programs, Canada has over a dozen solvent abuse treatment centres spread across the country[46]. These centres operate under a continuum of interventions, including prevention, early intervention, residential treatment and environmental deterrence. Furthermore, evidence suggests IDU with a solvent use background have a "specific course of addiction"[39], often with much more detrimental outcomes, and a particular intransigency to treatment[39,47]. This "deviant group within a deviant group" has been recognized since the late 1970s[47], but is still poorly understood, relative to other IDU groups.

Despite the link observed between solvent use and IDU, and the disproportionate burden of both solvent abuse and STI/BBP infection in Aboriginal populations, there is little published research on solvent use among Aboriginal IDU. We therefore undertook this study to examine the association between solvent use and socio-demographic and drug-related risk factors in Aboriginal IDUs in Manitoba, Canada. We were also interested in examining the relationship between solvent use and injection of other types of illicit substances, as well as being infected with a BBP (i.e., HIV and HCV).

## Methods

### Study setting and survey instrument

The study setting and survey instrument have been described previously[48-50]. This was a cross-sectional survey of IDU in Winnipeg, Manitoba, Canada (pop. 675,000) conducted from December 2003 to September 2004. Recruitment was advertised at local community health centres, meeting places (as identified by key informants) and word-of-mouth. Eligibility criteria included self-reported use of illicit injection drugs in the 6-month period prior to interview and having an age of 15 years or more. Potential participants made telephone contact with the study nurse, who administered all surveys in-person. Interviews took place in a private setting of the participant's choosing. An honorarium was provided to all study participants providing written or oral consent. The questionnaire was divided into three sections. The first section consisted of questions based on the respondent's own characteristics, the second elicited information on the respondent's egocentric network (i.e., the people with whom the respondent had regular contact with), while the third section asked questions on the respondent's IDU risk network. The first section was of primary interest for this study. The study design was approved by the Health Research Ethics Board of the University of Manitoba and the Winnipeg Regional Health Authority Research Review Committee.

### Measures

The outcome measure in this study was a binary variable describing solvent use, which was derived from a positive answer for "Gasoline/Solvents" to the survey item "In the last 6 months, which of the following drugs have you used without injecting?" The study sample of IDUs was subsetted to only individuals who self-identified as Aboriginal, and included those who identified as 'First Nations' or 'Metis'. Variables were grouped into four categories: socio-demographic, injection-related behaviours, other drug use and BBP status. Socio-demographic variables included: age, which was categorized as 15-29, 30-39, and 40 years or more; education, which was coded as 'dropped out less than grade 12' or 'grade 12 or higher'; and place of birth, which was coded as 'born inside Manitoba' or 'born elsewhere'. Injection-related behaviours included: locales where drugs were injected (in the last 6 months), and this list included their own house, a family members' or friends' residences, an empty house, a shelter/hostel, hotel, shooting gallery and on the street; sharing needles (ever and in the last 6 months); sharing other injection equipment; injecting someone as a service; injecting someone as a favour; and ease of obtaining needles. The time frame for the last four questions was 6 months.

Participants were asked which drugs they injected most frequently and finally, in terms of BBP infection, HIV and HCV status was assessed using venous blood samples, tested at Cadham Provincial Laboratory (Winnipeg, MB). Specimens were screened for HCV and HIV with AxSYM HCV (Abbott, Mississauga, ON) and AxSYM HIV1/2 gO (Abbott, Mississagua, ON), respectively. Presumptive positives were confirmed for HCV with Chiron HCV 3.0 RIBA (Ortho-Clinical Diagnostics, Markham, ON). Presumptive HIV positive specimens were confirmed by western blot (BioRad, Montreal, QC).

### Statistical methods

Associations between solvent use and variables of interest were assessed using χ^2 ^tests. Variables that were significant at the p < .20 level were included in multivariable logistic regression analysis. A parsimonious model was desired, so therefore, with the exception of sex (which was forced into the model to adjust for its effects), a backwards stepwise regression procedure was used to eliminate variables that were not significant at the p < .05 level. Odds ratios (OR) and their 95% confidence intervals (95% CI) are reported for univariate and multivariable analyses. Multicollinearity of the final model was assessed using VIF and tolerance statistics. Stata version 9 was used in performing all analyses[51].

## Results

A total of 272 IDU identified as Aboriginal. An additional 6 that identified as transgendered were excluded from the analyses due to small numbers, leaving a total sample size of 266. Overall, 44 (16.5%) of the study sample reported solvent use in the last 6 months. Table 1 displays a comparison of characteristics of solvent and non solvent-using IDU. Broadly speaking, the two groups differed significantly, at least at the p < .05 level, by age, injection locations, injection risk behaviours, type of drugs injected and BBP status (Table 1).

**Table 1:** Characteristics of 266 Aboriginal IDU by solvent-use status, Winnipeg Manitoba

	Solvent use status; no. (%)			
	Users (n = 44)	Non-users (n = 222)	Odds Ratio (95% CIs)	*P*
*Socio-Demographic*				
Age				
15-29	21(47.7)	51(22.4)	*Ref*	**<.001**
30-39	17(38.6)	86(37.7)	0.48 (0.23,0.99)	
40+	6(13.6)	91(39.9)	0.16 (0.07,0.42)	
*Mean (SD)*	*31.6(7.5)*	*36.3(9.1)*		
Female	25(56.8)	117(52.7)	1.18 (0.62,2.27)	.617
Born outside province	5(11.4)	37(16.7)	0.64 (0.24,1.73)	.362
At least grade 12	38(86.4)	169(76.1)	2.35 (0.90,6.15)	.135
*Injection Locations*				
Own House	27(61.4)	121(54.5)	1.33 (0.68,2.57)	.403
Family House	14(31.8)	32(14.4)	2.71 (1.32,5.79)	**.005**
Friend's House	37(84.1)	158(71.2)	2.14 (0.91,5.05)	.077
Empty House	10(22.7)	22(9.9)	2.67 (1.16,6.14)	**.017**
Shelter/Hostel	0(0.0)	6(2.3)	n/a	.277
Hotel	28(63.6)	95(42.8)	2.34 (1.20,4.57)	**.011**
Shooting Gallery	15(34.1)	35(15.8)	2.76 (1.35,5.66)	**.004**
Street	18(40.9)	56(25.2)	2.05 (1.05,4.02)	**.034**
*Injection Risk Behaviours*				
Share needles (Ever)	25(56.8)	104(46.9)	1.49 (0.78,2.87)	.227
Share needles (6 months)	15(34.1)	27(12.2)	3.74 (1.78,7.85)	**<.001**
Share injection equipment	13(29.6)	72(32.4)	0.87 (0.43,1.77)	.708
Inject someone as service	9(20.5)	62(28.2)	0.66 (0.29,1.44)	.291
Inject someone as favour	19(43.2)	97(44.1)	0.96 (0.50,1.85)	.912
Ease of obtaining needles	34(77.3)	188(84.7)	0.61 (0.28,1.36)	.227
*Drugs Injected*				
Cocaine	21(47.7)	152(68.5)	0.42 (0.22,0.81)	**.008**
Talwin & Ritalin	38(86.4)	78(35.1)	11.69 (4.73,28.87)	**<.001**
Morphine	7(15.9)	63(28.4)	0.48 (0.20,1.12)	.086
Heroin	0(0.0)	18(8.1)	n/a	--
Amphetamines	0(0.0)	4(1.8)	n/a	--
Methadone	0(0.0)	9(4.1)	n/a	--
Crack	4(9.1)	61(27.5)	0.26 (0.09,0.77)	**.010**
Crystal Methamphetamine	1(2.3)	9(4.1)	0.55 (0.07,4.46)	.570
Dilaudid	3(6.8)	23(10.4)	0.63 (0.18,2.21)	.470
Oxycontin	1(2.3)	6(2.7)	0.84 (0.10,7.13)	.871
*BBP Status*				
HIV *(n = 233)*	7(17.5)	16(8.3)	2.34 (0.90,6.15)	.082
Hepatitis C *(n = 238)*	33(80.5)	109(55.3)	3.33 (1.46,7.58)	**.004**

### Socio-demographic, injection-related and BBP status characteristics

Specifically, solvent-using IDU tended to be younger in age (p < .001) with an average age of 31.6 years (SD: 7.5), compared to non-solvent-using IDU, who averaged 36.3 years of age (SD: 9.1). Solvent users were more likely to have reported injecting in a family house (OR: 2.71; 95%CI: 1.32,5.79), empty house (OR: 2.67; 95%CI: 1.16,6.14), hotel (OR: 2.34; 95%CI: 1.20,4.57), shooting gallery (OR: 2.76; 95%CI: 1.35,5.66) and on the street (OR: 2.05; 95%CI: 1.05,4.02). Solvent users were more likely to have reported sharing needles in the last 6 months (OR: 3.74; 95%CI: 1.78,7.85). In terms of the most frequent drugs injected, solvent users were more likely to report Talwin & Ritalin injection (OR: 11.69; 95%CI: 4.73,28.87), while less likely to report cocaine (OR: 0.42; 95%CI: 0.22,0.81) and crack (OR: 0.26; 95%CI: 0.09,0.77) injection. No solvent users reported heroin, amphetamines or methadone as their most frequently injected drug. Finally, solvent users were more likely to be HCV positive (OR: 3.33; 95%CI: 1.46,7.58). Solvent users were more likely to be HIV positive than their non-solvent using counterparts (17.5% versus 8.3%), but this was not statistically significant at the p < .05 level (p = .076).

### Multivariable analysis

After backwards elimination, the following variables remained in the final logistic regression model (Table 2): HCV status (p = .016), sharing needles in the last 6 months (p = .048), Talwin & Ritalin injection (p < .001) and age (p < .001), adjusted for sex. All variables remained significant if sex was removed from the model.

**Table 2:** Adjusted Odds Ratios, Multivariable Logistic Regression of Predictors of Solvent Use, Aboriginal IDU, Winnipeg Manitoba

	Odds Ratio (95% CIs)	Standard Error	*p*
Hepatitis C	3.52 (1.27,14.68)	1.85	**.016**
Share needles (last 6 months)	2.61 (1.01,6.78)	1.27	**.048**
Talwin & Ritalin injection	9.97 (3.77,26.34)	4.94	**<.001**
Age (per year increase)	0.91 (0.86,0.96)	0.02	**<.001**
Female	0.80 (0.35,1.81)	0.33	.587

## Discussion

This study examined the association between solvent use in Aboriginal IDU and socio-demographic factors, drug-related risk factors, use of other illicit substances and BBP infection. We found that after adjusting for other variables including sex, solvent use was significantly associated with Talwin & Ritalin injection, HCV status and age in this population.

Some important limitations of the study should be stated at the outset. First and foremost, ours was a cross-sectional study, and a causal linkage between solvent use and injection drug use cannot be inferred from the data. Although both likely share determinants, our data are insufficient to establish causality. Aboriginal individuals in Canada face a combination of socially and structurally determined vulnerabilities, including high rates of entrenched poverty, unemployment, homelessness and sexual and physical abuse[2,52,53]. Many of these factors stem from a history of colonization, oppression, systemic racism and discrimination in Canadian society and have resulted in Aboriginal Canadians having unequal access to a variety of resources [2,54]. Thus, the perniciousness of both solvent and injection drug use within Aboriginal populations is more likely a result of these determinants. Second, solvent use was measured broadly. The measure used was not precise enough to discriminate between chronic and casual use. Similarly, different types of solvents were not captured in this study. Third, since a sampling frame was not possible to construct for this marginalized and hidden population, the sample was not randomly generated and may not be representative of Aboriginal IDUs in other settings, or in Winnipeg. Fourth, social desirability bias, or high non-response rate is always an issue with self-reported data; however, it is likely that this would have served to underestimate associations toward the null. Finally, the sample size was relatively small and thus may have not had power to detect significant findings.

Previous studies in Winnipeg have reported Talwin & Ritalin injection as being strongly associated with both Aboriginal ethnicity[50,55] and high HCV prevalence[49]. That HCV infection is three times more likely in the population of solvent-using Aboriginal IDU, after controlling for Talwin & Ritalin injection and risky injection practices, strongly suggests the existence of pockets of higher risk even amongst an already high-risk subpopulation[39,47]. It was also demonstrated that these qualitatively distinct 'higher-risk' groups can be distinguished when both injectable and non-injectable drug use is considered.

The relatively low prevalence of both HIV and HCV among IDU in our geographic setting has motivated researchers to ask what role, if any, public health responses in Winnipeg may have contributed to lower prevalence[56]. Both HIV and HCV prevalence in the subset of solvent-using IDU are relatively higher than other IDU in our sample; and at 18% and 81% respectively, are in closer alignment with the prevalence observed in other jurisdictions[57,58]. This dichotomy in prevalence reinforces the exceptionally high risk faced by solvent-using IDU, and their real or potential ability to be missed by what otherwise may be an effective public health response. This higher-risk group is particularly relevant given the recent attention paid to especially high rates of HIV in Aboriginal populations in central Canada[11,12], and serve to illustrate that BBP epidemics in Canada are not homogeneous.

Solvent use is an issue where there are no easily-identifiable solutions[23,24]. Solvent users are at the bottom of a drug-using hierarchy, in terms of perception by other substance users and practitioners, and by the sheer volume of their social and personal challenges[29,39,42,47]. Thus, given the already difficult lifestyle and behavioural issues related to injection drug use[58,59], a *combination *of solvent use and injection drug use within Aboriginal populations may present considerable, and specific challenges for treatment[39,47]. For example, although there is well-established literature on the effectiveness of harm-reduction efforts such as needle-exchange programs in curtailing the spread of BBPs[60,61], the constituents of an equivalent and appropriate harm reduction strategy for solvent users have not been well articulated in the literature [24], although practical advice may include using solvents in groups, and using clean rags or sponges. As well, outreach efforts to these populations may be unduly hampered by the considerable stigma attached to chronic solvent use. Similar to recent Canadian research demonstrating that IDU who also smoked crack cocaine were at higher risk of HIV seroconversion[10], perhaps an especially chaotic lifestyle is contributing to the higher HCV prevalence in our solvent-using subpopulation.

### Understanding outlier populations

As Kuller has suggested that understanding epidemics in "outlier" populations may have substantial benefits in unpacking transmission dynamics in more mainstream populations[62], a deeper examination of this, and similar subpopulations is warranted. Thus, we submit that understanding the exogenous factors that contribute to solvent use in IDU may result in better understanding of marginalized subpopulations in general, particularly with respect to understanding the trajectory of use[63]. For example, it has been recognized that solvent use is typically a group activity[23,24,26]. The natural consequence is the tendency to form closed networks[64], in this case comprised of fellow solvent-using IDU. This may be particularly true in our study population of Aboriginal IDUs, since individuals have been shown to form more cohesive structures according to ethnicity[65]. At the same time, the near ubiquity and accessibility of sources of solvents and inhalants is clearly a key contributor to their abuse[66]. Recent programs that seek to address solvent use in adolescent Aboriginal Canadians through improving individual-level coping strategies recognize that without multi-level support structures (e.g. family, community, environment) in place, individual recovery is likely to fail[25]. Other researchers have found that strong peer group sanctions against solvent use, in concert with messages concerning the dangers of solvent use were protective against lifetime and current use of solvents[23]. Thus, finding ways to identify and engage with solvent users and their peers may have application with other hidden and marginalized populations. Along this line, some authors have suggested that solvent use may be a marker for an inherently more challenging type of substance user[19,45]. Thus, it may be useful to understand to what extent the actual *choice *of solvent use is a proxy for characteristics that distinguish the most marginalized of subpopulations. Understanding the populations that become chronic abusers of easy-to-obtain substances (such as solvents) may help to facilitate a more general understanding of subpopulations that have proven to be intractable to treatment.

The fact that solvent use clusters around Talwin & Ritalin injection suggests two other interesting areas for future research. First, other authors have demonstrated the advantages of understanding IDU from a poly-injection drug use perspective[67]. Here, we have demonstrated the practicality of examining IDU in their use of both injection and non-injection drugs. At the treatment level, this perspective highlights the importance of treating two or more qualitatively distinct addictions concurrently[68,69]. For example, Stenbacka et al. demonstrated that opiate-injecting IDU undergoing methadone maintenance therapy (MMT) were more likely to relapse if they had co-occurring alcohol abuse issues[69]. Secondly, the clustering of solvent and Talwin & Ritalin use suggests that the use of either is driven, to a certain degree, by opportunism. Although our data cannot provide a definitive answer, it would be useful to know under what circumstances IDU resort to inhaling solvents. Assuming inhalation is their 'fallback' method, and philosophically similar to MMT, perhaps a reliable supply of other injectable or non-injectable drugs would deter this subpopulation of IDU from using solvents, and thus prevent some of the more serious neurological and cognitive deficits associated with long-term chronic use[70,71].

## Conclusion

In conclusion, although addressing social or peer group norms has long been advocated as part of an effective prevention and treatment strategy for IDU, perhaps structural-level interventions are especially indicated for solvent-using Aboriginal IDU. At a time when rates of HIV and other BBPs are escalating in Canadian Aboriginal populations, studies like this one can help inform targeted strategies, as well as motivate harm reduction research in very marginalized populations. The strong socially-constructed vulnerabilities of Aboriginal populations, the illegality of injection drug use, the obduracy of solvent use to traditional regulation and control, and the extreme marginalization of solvent users may be interacting to create a 'perfect storm' for those IDU already infected, and those at high risk for infection to slip through the cracks in public health systems.

## Competing interests

The authors declare that they have no competing interests.

## Authors' contributions

SS was responsible for conceptualization of the study, analysis, interpretation of data and writing of the manuscript. KD made substantial contributions to data analysis and interpretation and revised the manuscript critically, and made important intellectual contributions to the manuscript. AJ and JW conceived the study, acquired the data and helped draft the article, as well as revised it critically. All authors have given final approval for this version of the manuscript.
